# Genomic Analysis of Carotenoid and Vitamin E Biosynthetic Pathways in the Extremophilic Red Alga *Cyanidioschyzon merolae*

**DOI:** 10.3390/antiox14111303

**Published:** 2025-10-30

**Authors:** Yuanyuan Hui, Dexin Lyu, Na Huang, Shan Luo, Libao Zheng, Linyuan Zheng, Chuanming Hu, Li-En Yang, Pengfu Li, Shan Lu, Yinyin Deng

**Affiliations:** 1 School of Life Sciences, Nanjing University, Nanjing 210023, Chinadz1930003@smail.nju.edu.cn (N.H.); pengfuli@nju.edu.cn (P.L.); 2 Key Laboratory of Saline-Alkali Vegetation Ecology Restoration of the Ministry of Education, College of Life Science, Northeast Forestry University, Harbin 150040, China; 3 Jiangsu Marine Fisheries Research Institute, Nantong 226007, Chinalovenori@yeah.net (L.-E.Y.)

**Keywords:** antioxidants, carotenoid, *Cyanidioschyzon merolae*, metabolic pathway, red alga, vitamin E

## Abstract

*Cyanidioschyzon merolae*, an extremophilic unicellular red alga thriving in acidic hot springs at temperatures of 40–56 °C and pH 0.5–4.0, faces extreme oxidative stress conditions. This study presents a comprehensive genomic analysis of the carotenoid and vitamin E biosynthetic pathways, which are essential for antioxidant defense in this organism. Through comparative genomics using *Arabidopsis thaliana* sequences as queries, we identified and characterized genes encoding key enzymes involved in their metabolism. Our analysis reveals that *C. merolae* exclusively utilizes the methylerythritol-4-phosphate (MEP) pathway for isoprenoid biosynthesis and lacks a complete mevalonate (MVA) pathway. We identified eleven genes involved in terpenoid metabolism and seven genes specifically for carotenoid biosynthesis. Pigment analysis confirmed a streamlined carotenoid profile consisting solely of β-carotene, β-cryptoxanthin, and zeaxanthin, lacking the entire β,ε-branch and part of the β,β-branch. The complete tocopherol biosynthetic pathway produces exclusively α-tocopherol. The absence of the β,ε-carotenoid branch and the exclusive production of α-tocopherol demonstrate metabolic streamlining while maintaining antioxidant efficacy. These findings provide molecular blueprints for biotechnological applications, enabling targeted strategies to enhance antioxidant production through pathway optimization and metabolic engineering, while offering insights into developing stress-tolerant organisms and enhancing nutritional content in crops.

## 1. Introduction

Algae represent one of the most diverse and promising groups of organisms for sustainable bioresource development [1]. They offer exceptional versatility in producing high-value compounds, including proteins, lipids, carbohydrates, pigments, and bioactive molecules, while addressing global challenges related to food security, renewable energy, and environmental sustainability [2,3]. Microalgae such as *Chlorella* and *Spirulina* have already established themselves in the production of essential amino acids and vitamins, while *Dunaliella salina* serves as the primary commercial source of natural β-carotene, and *Haematococcus pluvialis* produces the potent antioxidant astaxanthin [4,5,6,7]. Beyond nutraceuticals, algae exhibit great potential for biofuel production, carbon dioxide sequestration, wastewater treatment, and the synthesis of pharmaceutical compounds [8,9]. Their autotrophic nature, rapid growth, minimal land requirements, and ability to thrive in non-arable environments make algae particularly attractive for sustainable biotechnology applications that do not compete with traditional agriculture.

Over the course of evolution, some algae have developed unique adaptations to extreme environments. For example, the unicellular red alga *Cyanidioschyzon merolae* was first isolated from an acidic hot spring and can survive under extreme conditions, including temperatures ranging from 40 °C to 56 °C and pH levels of 0.5 to 4.0 [10,11]. This unique feature suggests that *C. merolae* can serve as a robust chassis for bioproduction. The extreme conditions for its optimal growth effectively reduce the need for contamination management. Notably, like other photosynthetic organisms, *C. merolae* naturally produces high-value antioxidants, including provitamin A carotenoids (β-carotene, β-cryptoxanthin, and zeaxanthin) and α-tocopherol (vitamin E), compounds of significant commercial value in the pharmaceutical, nutraceutical, and cosmetic industries [12,13].

Unlike most eukaryotes, *C. merolae* possesses one of the smallest genomes, a single chloroplast, and a single mitochondrion [10]. The compact genome and simple cell structure enable the elucidation and further modification of its relatively simple metabolic network for the production of high-value products, without interference from multiple competing metabolic branches, as is often observed in green algae and higher plants.

The availability of complete genome sequences serves as a foundational resource that revolutionizes our ability to identify and characterize the complete sets of genes involved in specific metabolic pathways. Homologous genes can be identified through sequence similarity searches against well-characterized model organisms. This genomic foundation is particularly critical for non-model organisms with limited prior research, such as extremophilic algae. Furthermore, complete genome sequences are indispensable for developing genetic tools, designing targeted knockouts or overexpression systems, and applying synthetic biology approaches to enhance or redirect metabolic flux toward desired products.

Both carotenoids and vitamin E are essential antioxidant compounds synthesized in plastids, and geranylgeranyl diphosphate (GGPP) produced via the plastidial 2-C-methyl-D-erythritol 4-phosphate (MEP) pathway serves as a common substrate for their biosynthesis [14]. While carotenoids and vitamin E are crucial for human health, most animals, including humans, are unable to synthesize these essential nutrients and must rely on dietary sources for their intake [15]. Although metabolic pathways for synthesizing carotenoids and vitamin E have been extensively studied in higher plants, where their constituent profiles and corresponding genes are relatively conserved, very limited information is currently available in algae, which display significant diversity [16,17]. For example, Euglenophyta contain diadinoxanthin and diatoxanthin, Dinophyta (dinoflagellates) are characterized by the presence of peridinin, and some classes of Heterokontophyta have fucoxanthin as their unique carotenoid [18,19]. In addition to these examples, *C. merolae*, as a member of the most primitive lineage of red algae, is able to synthesize only β-carotene, β-cryptoxanthin, and zeaxanthin, which represent the simplest repertoire of carotenoids among all eukaryotic photosynthetic organisms [20].

Although the genome of *C. merolae* has been sequenced, there are only limited reports on its metabolic pathways [20]. This study focuses specifically on genes involved in carotenoid and vitamin E biosynthesis. By identifying these genes and outlining potential metabolic branches, we establish a foundation for transforming *C. merolae* from a biological curiosity into a tractable biotechnological platform with predictable and modifiable metabolic capabilities.

## 2. Materials and Methods

### 2.1. Material and Growth Conditions

*C. merolae* strain 10D was a gift from Dr. Jianren Shen. The alga was cultivated in 2× Allen’s medium (pH 2.5) at 42 °C under 55 μmol photon m^–2^ s^–1^ and a 12 h/12 h light/dark regime [21]. The culture was shaking at 100 rpm. Cells were harvested by centrifugation at 8000× *g* for 5 min for chemical analysis. Three batches of cultivated cells were used as repeats for chemical analysis below.

### 2.2. Bioinformatics Analysis

We used the tblastx algorithm and functionally identified sequences of the model plant *Arabidopsis* *thaliana*’s and the yeast *Saccharomyces cerevisiae*’s genes as queries. Sequence similarities of the *C. merolae* genes with their *A. thaliana* and *S. cerevisiae* homologs were calculated using the online sequence alignment tool (https://en.vectorbuilder.com/tool/sequence-alignment.html, accessed on 1 May 2025). All sequence information is presented in Table 1. The *C. merolae* genome sequence was from http://merolae.biol.s.u-tokyo.ac.jp (accessed on 1 May 2025).

**Table 1:** Genes encoding enzymes involved in carotenoid and vitamin E metabolism in *C. merolae*.

Gene ^1^	*C. merolae* Gene ID	*A. thaliana*		*S. cerevisiae*	
		Homolog ID	Similarity	Homolog ID	Similarity
*DXS*	CMF089C	At4g15560	67.17%	-	-
*DXR*	CMG148C	At5g62790	64.77%	-	-
*MCT*	CMH115C	At2g02500	54.30%	-	-
*CMK*	CMS444C	At2g26930	50.84%	-	-
*MDS*	CMT435C	At1g63970	29.21%	-	-
*HDS*	CML284C	At5g60600	38.39%	-	-
*HDR*	CMJ152C	At4g34350	68.67%	-	-
*IDI*	CMB062C	At3g02780	55.31%	BK006949.2	48.45%
*HMGS*	CMM189C	At4g11820	50.64%	NM_001182489.1	51.73%
*AACT1*	CMA042C	At5g47720	51.26%	BK006942.2	60.22%
*AACT2*	CME087C	At5g48230	62.39%	BK006949.2	48.33%
*AACT* *3*	CMR380C	At5g47720	21.54%	BK006942.2	23.77%
*FPPS*	CMM269C	At4g17190	63.21%	BK006943.2	61.22%
*GGPPS*	CMK058C	At4g36810	61.27%	BK006936.2	21.32%
*PSY*	CMM166C	At5g17230	46.95%	-	-
*PDS*	CMK151C	At4g14210	68.72%	-	-
*ZDS*	CMT061C	At3g04870	63.71%	-	-
*ZISO*	CMQ364C	At1g10830	47.80%	-	-
*CRTISO*	CMN268C	At1g06820	52.75%	-	-
*LCYB*	CMK050C	At3g10230	53.44%	-	-
*CrtR*	CMV041C	-	-	-	-
*HPPD*	CMI063C	At1g06570	50.31%	-	-
*GGR*	CMJ154C	At1g74470	71.46%	-	-
*VTE1*	CML326C	At4g32770	41.07%	-	-
*VTE2.1*	CMN202C	At2g18950	55.88%	-	-
*VTE2.2*	CMS413C	At2g18950	47.86%	-	-
*VTE3*	CMD011C	At3g63410	62.83%	-	-
*VTE4*	CMT560C	At1g64970	43.03%	-	-
*VTE5*	CMR252C	At5g04490	39.42%	-	-
*VTE6*	CMS030C	At1g78620	52.28%	-	-

^1^ Full names of the genes are provided in the legend of Figure 1.

**Figure 1:**
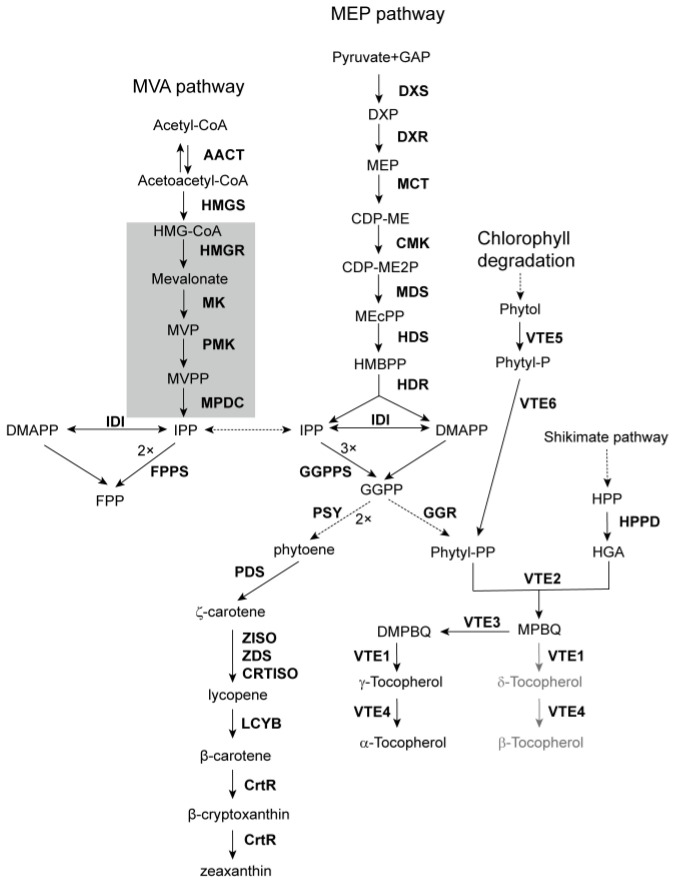
Carotenoid and tocopherol biosynthetic pathways in *C. merolae*. The shadowed area indicates the metabolic steps that are absent in *C. merolae*. Dashed arrows indicate multiple steps. Gray arrows and products indicate undiscovered metabolic steps. Abbreviations of the metabolites are: GAP, glyceraldehyde 3-phosphate; DXP, 1-deoxy-D-xylulose 5-phosphate; MEP, 2-*C*-methyl-D-erythritol 4-phosphate; CDP-ME, 4-diphosphocytidyl-2*C*-methyl-d-erythritol; CDP-ME2P, 4-cytidinediphospho-2-*C*-methylerythritol 2-phosphate; MEcPP, 2-*C*-methyl-D-erythritol 2,4-cyclo-diphosphate; HMBPP, 4-hydroxy-3-methylbut-2-enyl diphosphate; HMG-CoA, 3-hydroxy-3-methylglutaryl-CoA; MVP, mevalonate phosphate; MVPP, mevalonate diphosphate; IPP, isopentenyl diphosphate; DMAPP, dimethylallyl diphosphate; GGPP, geranylgeranyl diphosphate; HPP, 4-hydroxyphenylpyruvate; HGA, homogentisic acid; MPBQ, 2-methyl-6-phytyl-1,4-benzoquinol; DMPBQ, 2,3-dimethyl-6-phytyl-1,4-benzoquinone (DMPBQ); and for the enzymes (in bold) are: AACT, acetyl-CoA acetyltransferase; HMGS, HMG-CoA synthase; HMGR, HMG-CoA reductase; MK, mevalonate kinase; PMK, phosphomevalonate kinase; MPDC, mevalonate diphosphate decarboxylase; DXS, DXP synthase; DXR, DXP reductoisomerase; MCT, MEP cytidylyltransferase; CMK, CDP-ME kinase; MDS, MEcPP synthase; HDS, HMBPP synthase; HDR, HMBPP reductase; IDI, IPP/DMAPP isomerase; GGPPS, GGPP synthase; PSY, phytoene synthase; PDS, phytoene desaturase; ZISO, ζ-carotene isomerase; ZDS, ζ-carotene desaturase; CRTISO, carotene isomerase; LCYB, lycopene β-cyclase; CrtR, carotene hydroxylase; GGR, geranylgeranyl reductase; VTE5, phytol kinase; VTE6, phytyl phosphate kinase; HPPD, 4-hydroxyphenylpyruvate dioxygenase; VTE2, HGA phytyltransferase; VTE3, MPBQ methyltransferase; VTE1, tocopherol cyclase; VTE4, γ-tocopherol methyltransferase.

### 2.3. Metabolite Analysis

For terpenoid analysis, pelleted algal cells were extracted with methyl tert-butyl ether (MTBE). The sample was subjected to gas chromatography–mass spectrometry (GC–MS) analysis using a GC-MS-TQ8050 NX system (Shimadzu, Kyoto, Japan) and an HP-5MS (30 m × 0.25 mm; film thickness, 0.25 μm) column (Agilent, Santa Clara, CA, USA). Helium was used as the carrier gas. The GC oven temperature was programmed as follows: the initial temperature was 45 °C and held for 2 min, increased at a rate of 10 °C min^–1^ up to 250 °C and held for 5 min. The ion source temperature was set to 280 °C. The acquisition was made in scan mode (60 to 300 *m*/*z*).

Carotenoid detection is carried out according to the previous report [22]. In brief, to 100 mg pelleted cells, 400 μL 80% acetone, 250 μL ethyl acetate, and 250 μL distilled water were added sequentially, with a 30 s vortex after each addition to mix thoroughly. After centrifugation at 15,000× *g* for 10 min, the upper organic phase was collected for high-performance liquid chromatography (HPLC) analysis. An Agilent 1260 HPLC system (Agilent) with a Spherisorb ODS2 column (5 μm, 4.6 mm × 250 mm) (Waters, Milford, MA, USA) and a 2998 photodiode array detector (PDA, Agilent) was used. A 30 min gradient of ethyl acetate (0–66.7%) in mobile phase A (acetonitrile:water:trimethylamine = 9:1:0.01, *v*/*v*/*v*) at 35 °C was used.

For vitamin E analysis, 1 mL hexane containing 0.01% butylated hydroxytoluene (BHT) as an antioxidant was mixed thoroughly with 100 mg pelleted algal cells by vortex. The extract was left at room temperature for 20 min and then centrifuged at 4000× *g* for 10 min. The pellet was repeatedly extracted once. The upper organic phase was combined and dried under a stream of nitrogen. The dried samples were redissolved in 200 μL of methanol for HPLC analysis. An Agilent 1260 HPLC system equipped with an Eclipse Plus C18 (4.6 × 250 mm, 5 μm) column and a 2998 PDA was used for separation. The elution conditions were 100% mobile phase (methanol:water = 98:2) at 1 mL min^–1^, and the detection wavelength was 292 nm. The retention times of each peak were compared with those of authentic chemicals that were analyzed in parallel for identification [23,24].

For each batch of cultivated algal cells, three repeats were performed for each of the GC-MS assay of terpenoids, and the HPLC assay of carotenoids and vitamin E.

## 3. Results

### 3.1. Terpenoid Metabolism

Terpenoids represent the largest class of natural products, which are ubiquitously synthesized in all organisms, with more than 80,000 structures characterized to date [25]. Although the structures and functions of terpenoids are highly diverse, all of them share the same C5 isoprenoid precursors, isopentenyl diphosphate (IPP) and its isomer dimethylallyl diphosphate (DMAPP). In vascular plants, these precursors are synthesized through two parallel pathways: the cytosolic mevalonate (MVA) pathway and the plastidial MEP pathway [26]. However, algae display a great diversity in the operation of these two pathways. For example, while green algae and most multicellular red algae exclusively use the MEP pathway, some unicellular red algae have been reported to possess both pathways [27].

Our comprehensive search of the *C. merolae* genome revealed a total of 13 genes involved in terpenoid biosynthesis, including all eight genes of the MEP pathway, the first 3 of the MVA pathway, and 3 short-chain prenyltransferases (PTs) (Table 1). For the MEP pathway, there is one gene copy for each of the following eight enzymes: (1) 1-deoxy-D-xylulose 5-phosphate (DXP) synthase (DXS), which catalyzes the condensation of glyceraldehyde 3-phosphate and pyruvate to produce DXP; (2) DXP reductoisomerase (DXR), which converts DXP to MEP as the first rate-limiting step in terpenoid metabolism; (3) MEP cytidyltransferase (MCT), which converts MEP into 4-diphosphocytidyl-2-*C*-methyl-D-erythritol (CDP-ME); (4) CDP-ME kinase (CMK), which further phosphorylates CDP-ME into 4-cytidinediphospho-2-*C*-methylerythritol 2-phosphate (CDP-ME2P); (5) 2-*C*-methyl-D-erythritol-2,4-cyclo-diphosphate (MEcPP) synthase (MDS), which uses CDP-ME2P as a substrate to synthesize MEcPP; (6) 4-hydroxy-3-methylbut-2-enyl diphosphate (HMBPP) synthase (HDS), which converts MEcPP to HMBPP; (7) HMBPP reductase (HDR), which produces both IPP and DMAPP simultaneously, with a preference for IPP; and (8) IPP/DMAPP isomerase (IPI), which interconverts IPP and DMAPP to maintain their balance (Figure 1) [14].

Using *A. thaliana* genes of the MVA pathway as queries, we identified only three *C. merolae* homologous genes: two genes encoding acetyl-CoA acetyltransferases (AACTs), which catalyze the condensation of acetyl-CoA to acetoacetyl-CoA, and one gene encoding 3-hydroxy-3-methylglutaryl-CoA (HMG-CoA) synthase (HMGS), which catalyzes the biosynthesis of HMG-CoA as the first rate-limiting step of the pathway, as reported in vascular plants (Table 1, Figure 1) [28,29]. No homologous genes encoding enzymes beyond HMG-CoA in this pathway could be identified using the corresponding *A. thaliana* sequences as queries. However, considering the extreme evolutionary distance between these two organisms, the *C. merolae* homologs may have sequence similarities too low to be detected by the *A. thaliana* queries. For this reason, we used the annotated *HMGS*, *HMGR*, *MK*, and *MPDC* sequences from the green alga *Mesostigma viride*, which has been proposed as an ancient streptophyte, as queries for another round of blast search [28]. However, our BLAST (ver. 2.16.0) search identified only one *HMGS* gene from the *C. merolae* genome, which was identical to the one obtained using *AtHMGS* as a query (Table S1). Moreover, because the *M. viride* gene sequences deposited in GenBank are incomplete, the sequence similarity between *C. merolae* and *M. viride HMGS* homologs was slightly lower than that between the *C. merolae* homolog and *AtHMGS* (Table S1).

Because most of the algal genes related to terpenoid metabolism have been actually annotated or predicted using *A. thaliana* and other model land plants genes as queries [30,31,32], there is a possibility that *C. merolae* genes of the MVA pathway have an alternative origin. Therefore, we further used genes from the yeast *Saccharomyces cerevisiae* as queries to search the *C. merolae* genome. Again, no homologous genes beyond *HMGS* could be identified (Table 1). However, an additional *AACT* (*CmAACT3*) was identified, sharing 23.77% and 21.54% sequence similarities with its *S. cerevisiae* (BK006942.2) and *A. thaliana* (*At5g47720*) homologs, respectively (Table 1). Encouraged by this result, we further used *HMGR* sequences from a bacterium (*Pediococcus pentosaceus*, NC_008525.1) and an animal (*Mus musculus*, XM_146397.3) as queries. However, no *C. merolae* homolog could be identified from the genome (Table S1). Taken together, it is unlikely that *C. merolae* operates both MVA and MEP pathways as does another unicellular red alga, *Galdieria sulphuraria* [33]. Its IPP and DMAPP required for terpenoid biosynthesis are probably derived exclusively from the MEP pathway. According to the endosymbiosis theory, red algae evolved after an ancestral cyanobacterium operating the MEP pathway was engulfed by a eukaryotic host operating the MVA pathway [34]. Therefore, the ancestral red alga might have harbored both pathways, as reflected by modern *G. sulphuraria* and a few other unicellular red algae. Both *G. sulphuraria* and *C. merolae* have highly compact genomes with low gene numbers and are proposed to have experienced extensive gene reduction [35]. It is possible that different repertoires of genes were lost during the evolution of these two species, both of which belong to the most primitive red algal class Cyanidiophyceae.

PTs are a class of enzymes that catalyze the sequential condensation of different numbers of IPP molecules to one molecule of DMAPP, generating prenyl diphosphates with varying chain lengths. These enzymes are typically named after their respective products [36]. For instance, geranyl diphosphate (GPP, C10) synthase (GPPS) utilizes IPP and DMAPP at a 1:1 ratio to produce GPP, the immediate precursor of monoterpenoids; farnesyl diphosphate (FPP, C15) synthase (FPPS) utilizes a 2:1 ratio to produce FPP, a common substrate for synthesizing sesquiterpenoids and sterols (C30); and geranylgeranyl diphosphate (GGPP, C20) synthases (GGPPS) utilizes a 3:1 ratio to produce GGPP, a central intermediate shared among multiple downstream pathways, including those for diterpenoid products, tetraterpenoids (C40, e.g., carotenoids and their derivatives), and the side chains of chlorophylls and vitamin E. Sequence analysis has shown that GPPS and GGPPS share a high degree of similarity and are generally classified within the same enzyme family. In contrast, FPPS constitutes a distinct evolutionary lineage [37].

**Figure 2:**
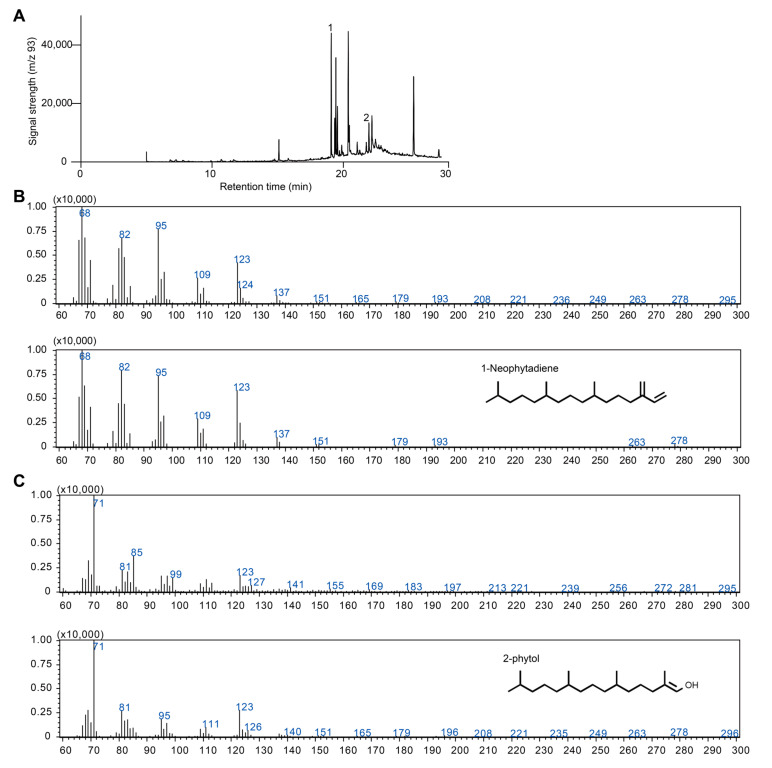
GC-MS analysis and identification of terpenoid compounds in *C. merolae*. (**A**) GC-MS profile of *C. merolae* extract. Only peaks 1 and 2 were identified as terpenoids. (**B**) Comparison of the mass spectrum of peak 1 (top) and the standard spectrum of 1-neophytadiene (bottom). (**C**) Comparison of the mass spectrum of peak 2 (top) and the standard spectrum of 2-phytol.

In our genome-wide analysis, we identified one gene homologous to the FPPS family and another belonging to the GPPS/GGPPS family (Table 1; Figure 1). The presence of both genes might suggest the capability of synthesizing monoterpenoids and sesquiterpenoids, and occasional reports have described such constituents in red algae [38,39]. However, several lines of evidence challenge these findings. First, the biosynthesis of monoterpenoids and sesquiterpenoids typically requires specific terpene synthases (TPSs) that utilize GPP and FPP as substrates [40]. Recent evolutionary analyses indicate that plant-type TPS enzymes only emerged after the divergence of land plants from their charophytic algal ancestors. Moreover, no microbial-type TPS homologs have been detected in the *C. merolae* genome [41,42,43]. In addition, previous studies revealed that members of the GPPS/GGPPS family in red algae are bona fide GGPPSs that produce only GGPP. The capability of these enzymes to generate GPP appears to have originated in early land plants such as bryophytes [37,44,45]. Taken together, these findings suggest that *C. merolae* is unlikely to synthesize mono- or sesquiterpenoid products, at least not through the canonical metabolic pathway observed in other plant lineages. To further confirm our conclusion, we cultivated *C. merolae* and extracted its volatile constituents for GC-MS analysis. From this analysis, only two diterpenoid compounds, 1-neophytadiene and 2-phytol, were identified, which appear to be generated from chlorophyll degradation (Figure 2).

### 3.2. Carotenoid Biosynthesis

**Figure 3:**
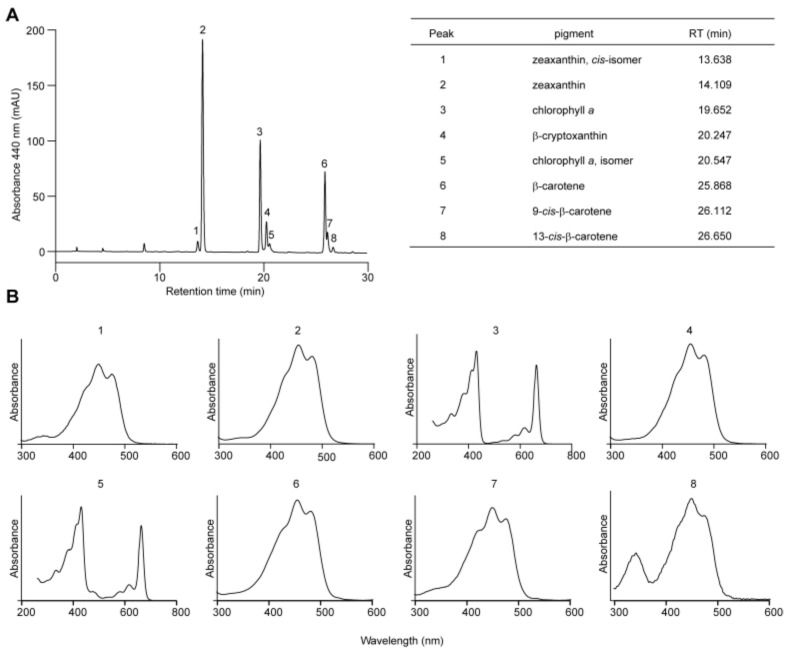
Pigment profile of *C. merolae*. (**A**) HPLC separation of pigments extracted from *C. merolae* culture. Retention times of numbered peaks (1–8) are provided. (**B**) UV-Vis absorption spectra for each numbered peak.

Carotenoid biosynthesis begins with the condensation of two molecules of GGPP by phytoene synthase (PSY) to form phytoene, which acts as the entry enzyme channeling GGPP flux into the carotenoid pathways (Figure 1). Vascular plants share a common carotenoid profile that includes constituents containing either two β-rings (the β,β-branch, e.g., β-carotene, zeaxanthin, antheraxanthin, violaxanthin, etc.) or one β- and one ε-rings (the β,ε-branch, e.g., α-carotene and lutein). Since the 1990s, the genes and enzymes involved in carotenoid biosynthesis in plants and microorganisms have been gradually elucidated [16]. Generally, phytoene undergoes two desaturation and two isomerization steps catalyzed sequentially by phytoene desaturase (PDS), ζ-carotene isomerase (ZISO), ζ-carotene desaturase (ZDS), and carotene isomerase (CRTISO), to produce linear lycopene (Figure 1). Lycopene represents the first branching point in carotenoid metabolism. It may be cyclized by lycopene β-cyclase (LCYB) alone, introducing two β-rings to both open ends to form β-carotene and its derivatives in the β,β-branch, or by LCYB together with lycopene ε-cyclase (LCYE), introducing one β- and one ε-ring to form α-carotene and its derivatives in the β,ε-branch (Figure 1). In rare species, lycopene can also be cyclized by LCYE alone to produce ε-carotene and its derivatives that have two ε-rings on both ends. Consistent with the previous report [20], our HPLC analysis revealed only β-carotene, zeaxanthin, and their metabolic intermediates in *C. merolae*, suggesting that it possesses the simplest carotenoid repertoire among photosynthetic eukaryotes, similar to that of the prokaryotic cyanobacteria (Figure 3).

From our search, we identified a total of seven homolog genes encoding enzymes for carotenoid biosynthesis in *C. merolae*, including PSY, PDS, ZDS, ZISO, CRTISO, LCYB, and a non-heme CrtR-type carotene hydroxylase (Table 1, Figure 1). Interestingly, whereas previous studies in red algae identified only a single gene for PDS and ZDS in *Porphyra umbilicalis*, we found two separate genes encoding these two enzymes in *C. merolae* (Table 1) [46]. We further performed a BLAST search of their homologs in other red algal species with complete genomes and identified homologs for both enzymes in most of them. The lack of additional homologous desaturase genes in the carotenoid pathway in *P. umbilicalis* may be due to a genome assembly issue.

LCYB has been previously characterized. Recent studies of other lycopene cyclases demonstrated a duplication and neofunctionalization of LCYE within red algae, parallelling a similar event in the green lineage [47]. The absence of the complete β,ε-branch of carotenoids is in agreement with the absence of LCYE in *C. merolae*. Conversion of β-carotene to zeaxanthin requires carotene β-hydroxylase, which is typically mediated by non-heme and/or P450-type enzymes in vascular plants. A P450-type enzyme was previously cloned and characterized as carotene β-hydroxylase from the multicellular red algal seaweed *P. umbilicalis*; however, no homolog was found in the *C. merolae* genome [46]. Although a gene encoding a CrtR-type hydroxylase was identified and cloned from *C. merolae*, its cognate protein has not yet been functionally characterized, leaving the mechanism of zeaxanthin formation unclear [20]. Nevertheless, the combination of zeaxanthin accumulation, the absence of the entire β,ε-branch, and a minimal β,β-branch strongly suggests that *C. merolae* represents a metabolically streamlined system for carotenoid biosynthesis. This minimal carotenoid network makes it an ideal chassis for metabolic engineering, offering a clean background for the heterologous production of high-value carotenoids such as capsanthin and astaxanthin, without interference from competing endogenous pathways.

### 3.3. Vitamin E Biosynthesis

Vitamin E comprises a family of lipid-soluble antioxidants, including tocopherols and tocotrienols, all of which share a homogentisic acid (HGA) aromatic head group derived from the shikimate pathway. The two classes are distinguished by their side chains: tocotrienols carry an unsaturated geranylgeranyl side chain, whereas tocopherols possess a saturated phytyl side chain. The geranylgeranyl side chain is derived directly from GGPP. The phytyl side chain can arise via two routes suppling phytyl diphosphate (Phytyl-PP): one through direct reduction of GGPP by geranylgeranyl reductase (GGR) as an extension of the MEP pathway, and the other through chlorophyll degradation, in which the released phytol is sequentially re-phosphorylated by two kinases, phytol kinase (namely VTE5 in *A. thaliana*) and phytyl phosphate kinase (VTE6) (Table 1, Figure 1) [48].

Key genes for vitamin E biosynthesis were initially cloned and characterized in *A. thaliana* and the cyanobacterium *Synechocystis* sp. PCC 6803 [17]. Besides VTE5 and VTE6 mentioned above, 4-hydroxyphenylpyruvate dioxygenase (HPPD) catalyzes the initial ring-cleavage steps leading to the production of HGA (Figure 1) [49]. HGA phytyltransferase (HPT/VTE2) and HGA geranylgeranyltransferase (HGGT) catalyze the condensation of HGA with Phytyl-PP or GGPP, producing 2-methyl-6-phytyl-1,4-benzoquinol (MPBQ) or 2-methyl-6-geranylgeranyl-1,4-benzoquinol (MGGBQ), respectively (Figure 1) [50]. MPBQ can be methylated to produce 2,3-dimethyl-6-phytyl-1,4-benzoquinone (DMPBQ) by methyltransferase (VTE3) [51]. Both MPBQ and DMPBQ can be catalyzed by a cyclase (VTE1) and a methylase (VTE4) to produce α-tocopherol and β-tocopherol, respectively (Figure 1) [24,52,53]. In parallel, similar reactions starting from MGGBQ produce α- and β-tocotrienols, respectively. Tocopherols are universal compounds in most higher plants, whereas tocotrienols occur primarily in the seeds of monocot species, such as barley, wheat, oat, rye, rice, palm tree, and coconut [54].

**Figure 4:**
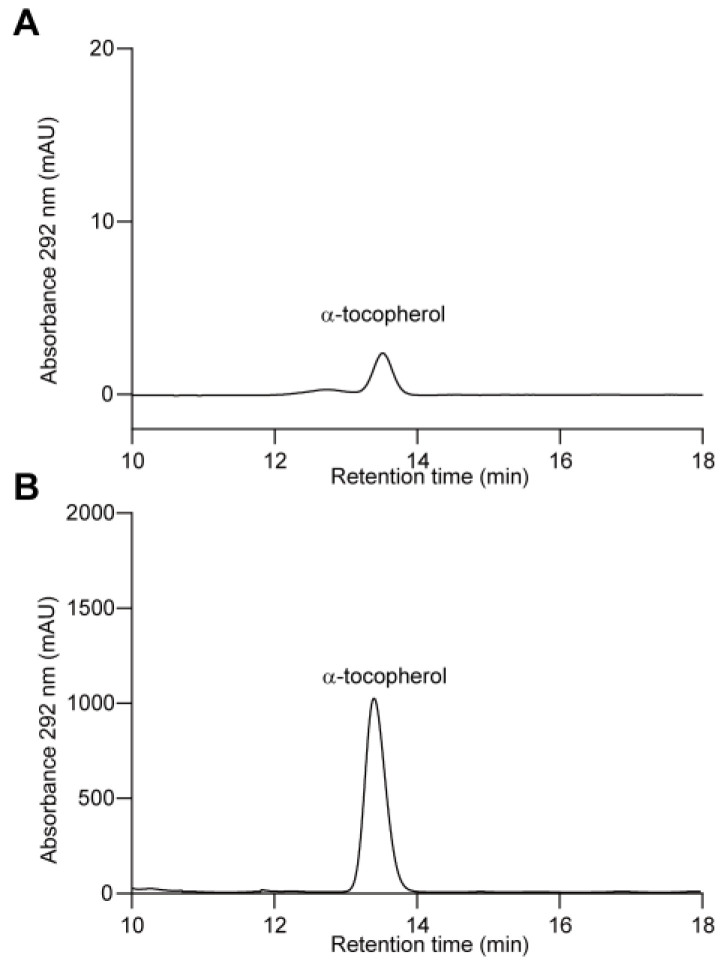
HPLC separation of vitamin E in *C. merolae*. (**A**) HPLC separation of vitamin E extracted from *C. merolae*. The absorbance at 292 nm was monitored. (**B**) A separation of authentic α-tocopherol in parallel with (**A**).

No previous studies have reported vitamin E in *C. merolae* before. In this study, we extracted vitamin E from cultivated algal cells and performed HPLC analysis. From our results, it was clear that only α-tocopherol could be detected, indicating that *C. merolae* solely uses the phytyl side chain (Figure 4 and Figure S1). However, two homologous copies of *VTE2* were identified in the *C. merolae* genome, in contrast to *A. thaliana*, which has a single copy of the *VTE2* gene (Table 1). It remains unclear how the two genes evolved and whether one of them was neofunctionalized to utilize GGPP for tocotrienol production [55]. In addition, the absence of β-tocopherol suggests that VTE1 preferentially utilizes DMPBQ as a substrate in *C. merolae*.

## 4. Discussion and Conclusions

The availability of complete genome sequences provides an unprecedented opportunity for metabolic engineering. Such a strategy has been successfully applied to a wide range of microbes, crops, medicinal and industrial plants, etc. However, identifying gene homologs in primitive red algae presents significant challenges compared to land plants. The bottleneck probably lies in the limited information on the genomics and molecular biology of red algae, compared with the extensively sequenced genomes and well-studied traits of model plants and major crops such as Arabidopsis, rice, and maize. In this study, our analysis revealed complete sets of genes for the biosynthesis of carotenoids and tocopherols, two major classes of antioxidants, as well as their upstream terpenoids, in *C. merolae*. Our analysis has the following three main findings.

First, *C. merolae* has an incomplete MVA pathway for terpenoid biosynthesis. The incomplete MVA pathway without enzymes beyond HMGS (Table 1) exemplifies metabolic efficiency through evolutionary gene reduction. By exclusively utilizing the MEP pathway, the alga avoids energy-intensive cytosolic steps, optimizing isoprenoid flux toward plastidial antioxidants under stressed conditions such as high temperature and low pH. This streamlining reduces metabolic redundancy, as seen in compact genomes of extremophiles, enabling rapid adaptation to oxidative bursts in its acidic hot spring habitat [14]. Moreover, in plants, HMG-CoA functions not only as a precursor for the mevalonate pathway but also as a reversible reservoir of acetoacetyl-CoA and acetyl-CoA [56,57,58]. It is currently unclear whether the incomplete MVA pathway in *C. merolae* participates in the redistribution of carbon flux.

Second, *C. merolae* can synthesize only a limited group of carotenoids. Our genome analysis failed to identify a gene homolog for LCYE, consistent with chemical analysis indicating the absence of lutein and other carotenoid species of the β,ε-branch (Table 1, Figure 3). Moreover, there are no homologous genes for ZEP and beyond in the *C. merolae* genome (Table 1). This also supports our result that zeaxanthin of the β,β-branch is the end product in the carotenoid biosynthetic pathway (Figure 3). Both the gene repertoire and carotenoid profile of *C. merolae* resemble those of prokaryotic cyanobacteria, reflecting the primitive nature of this unicellular red alga [18].

Third, α-tocopherol is the only vitamin E constituent in *C. merolae*. Enzymes like VTE2 (with promiscuous duplicates VTE2.1 and VTE2.2) and VTE3 exhibit mechanistic versatility. VTE2 catalyzes HGA–phytyl condensation, but its dual copies may allow substrate promiscuity, enabling further functional divergence toward handling GGPP for tocotrienol biosynthesis during evolution. Methylation of MPBQ by VTE3 ensures efficient DMPBQ formation, favoring a prompt metabolic flux from VTE2 to VTE1. This likely enhances pathway resilience by buffering against substrate fluctuations [17]. Functional assays could further elucidate these roles.

Similar to the promiscuous functions of genes for VTE2.1/2.2 and VTE3, the unresolved evolutionary relationship of AACT1/2 and PDS/ZDS with their homologs across different linages of photosynthetic organisms, as well as the puzzle surrounding the production of mono- and sesquiterpenoids, also merits further analysis.

This comprehensive identification of carotenoid and vitamin E biosynthetic genes in *C. merolae* provides an essential molecular blueprint for antioxidant synthesis that underpins its resilience in extreme niches. Simplified pathways (e.g., MEP-exclusive isoprenoids leading to zeaxanthin and α-tocopherol) minimize energy diversion and channel resources toward ROS scavenging. In acidic hot springs, these antioxidants may help stabilize membranes and protect photosystems from thermal and oxidative damage, enabling survival where other algae fail [10,11]. Insights gained from *C. merolae*’s streamlined yet efficient antioxidant machinery can guide efforts to enhance stress tolerance and nutritional value in agricultural and industrial crops, contributing to food security and sustainable agriculture under increasingly challenging environmental conditions. This study also offers strategies for advancing bioresource development and metabolic engineering applications in sustainable biotechnology. Targeted pathway optimization, gene overexpression, metabolic flux redirection, and rational synthetic biology design could help transform *C. merolae* into an efficient production platform for commercially valuable antioxidants.

## Data Availability

The original contributions presented in this study are included in the article/Supplementary Materials. Further inquiries can be directed to the corresponding authors.

## Supplementary Materials

The following supporting information can be downloaded at: https://www.mdpi.com/article/10.3390/antiox14111303/s1. Figure S1: HPLC separation of authentic standards of tocopherols in *C. merolae*. Table S1: Genes encoding enzymes in the MVA pathway in *C. merolae* identified using non-Arabidopsis queries.

## Abbreviations

The following abbreviations are used in this manuscript:

AACT	acetyl-CoA acetyltransferases
BHT	butylated hydroxytoluene
CDP-ME	4-diphosphocytidyl-2-C-methyl-D-erythritol
CDP-ME2P	4-cytidinediphospho-2-C-methylerythritol 2-phosphate
CMK	CDP-ME kinase
CRTISO	carotene isomerase
DMAPP	dimethylallyl diphosphate
DMPBQ	2,3-dimethyl-6-phytyl-1,4-benzoquinone
DXP	1-deoxy-D-xylulose 5-phosphate
DXR	DXP reductoisomerase
DXS	DXP synthase
FPP	farnesyl diphosphate
FPPS	FPP synthase
GC-MS	gas chromatography–mass spectrometry
GGPP	geranylgeranyl diphosphate
GGPPS	GGPP synthase
GGR	geranylgeranyl reductase
GPP	geranyl diphosphate
GPPS	GPP synthase
HDR	HMBPP reductase
HDS	HMBPP synthase
HGA	homogentisic acid
HGGT	HGA geranylgeranyltransferase
HMBPP	4-hydroxy-3-methylbut-2-enyl diphosphate
HMG-CoA	3-hydroxy-3-methylglutaryl-CoA
HMGS	HMG-CoA synthase
HPLC	high-performance liquid chromatography
HPPD	4-hydroxyphenylpyruvate dioxygenase
HPT	HGA phytyltransferase
IPI	IPP/DMAPP isomerase
IPP	isopentenyl diphosphate
LCYB	lycopene β-cyclase
LCYE	lycopene ε-cyclase
MCT	MEP cytidyltransferase
MDS	MEcPP synthase
MEcPP	2-C-methyl-D-erythritol 2,4-cyclo-diphosphate
MEP	2-C-methyl-D-erythritol 4-phosphate
MGGBQ	2-methyl-6-geranylgeranyl-1,4-benzoquinol
MPBQ	2-methyl-6-phytyl-1,4-benzoquinol
MTBE	methyl tert-butyl ether
MVA	mevalonate
PDA	photodiode array detector
PDS	phytoene desaturase
PSY	phytoene synthase
TPS	terpene synthase
ZDS	ζ-carotene desaturase
ZISO	ζ-carotene isomerase
