# Supplementary material for: Genomic Analysis of Carotenoid and Vitamin E Biosynthetic Pathways in the Extremophilic Red Alga Cyanidioschyzon merolae
Source: Antioxidants (Basel). 2025 Oct 30;14(11):1303. doi: 10.3390/antiox14111303 (PMC12649381; doi:10.3390/antiox14111303)
Supplement: Supplementary file 1 [file antioxidants-14-01303-s001.zip › Table S1.pdf]

Table S1. Genes encoding enzymes in the MVA pathway in *C. merolae* identified using non-*Arabidopsis* queries.

Gene	*C. merolae* Gene ID	Query		*A. thaliana*	
		ID	Similarity	homolog ID	Similarity
Mesostigma viride					
HMGS	CMM189	EF119231	45.26%	At4g11820	50.64%
HMGS	-	EF119234	-	At4g11820	-
HMGR	-	EF119235	-	At1g76490	-
HMGR	-	EF119232	-	At1g76490	-
MK	-	EF119236	-	At5g27450	-
MPDC	-	EF119233	-	At2g38700	-
MPDC	-	EF119237	-	At2g38700	-
Pediococcus pentosaceus					
HMGR	-	NC_008525.1			
Mus musculus					
HMGR	-	XM_146397.3			
