# Supplementary figures and images for: Genomic Analysis of Carotenoid and Vitamin E Biosynthetic Pathways in the Extremophilic Red Alga Cyanidioschyzon merolae
Source: Antioxidants (Basel). 2025 Oct 30;14(11):1303. doi: 10.3390/antiox14111303 (PMC12649381; doi:10.3390/antiox14111303)

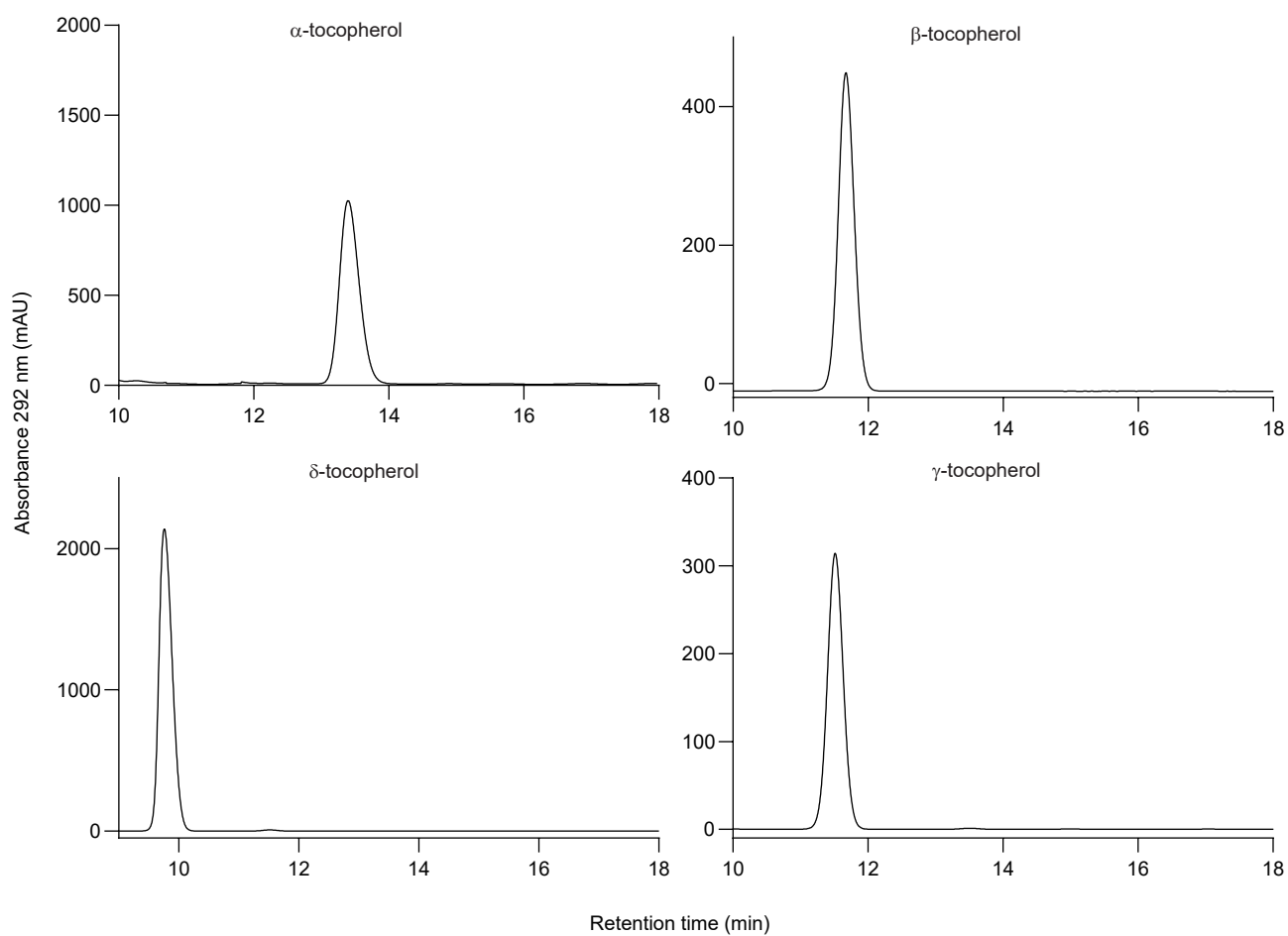

**Fig. S1. HPLC separation of authentic standards of tocopherols in *C. merolae*.**

Supplement: Supplementary file 1 [file antioxidants-14-01303-s001.zip › Supplementary Figure 1. HPLC analysis of tocopherol standard.pdf]
